# Physiological and transcriptomic analysis reveal the crucial factors in heat stress response of red raspberry ‘Polka’ seedlings

**DOI:** 10.3389/fpls.2023.1233448

**Published:** 2023-08-09

**Authors:** Juanjuan Guo, Ruiyu Zhang, Siqi Cheng, Ziqian Fu, Peng Jia, Haoan Luan, Xuemei Zhang, Guohui Qi, Suping Guo

**Affiliations:** College of Forestry, Hebei Agricultural University, Baoding, China

**Keywords:** red raspberry, seeding, heat stress, physiological analysis, transcriptomic analysis

## Abstract

With global climate warming, recurring extreme heat and high temperatures irreversibly damage plants. Raspberries, known for their nutritional and medicinal value, are in high demand worldwide. Thus, it is important to study how high-temperature stress (HTS) affects raspberries. The physiological and biochemical responses and molecular genetic mechanisms of raspberry leaves to different HTS treatments were investigated: mild high temperature at 35°C (HT35), severe high temperature at 40°C (HT40), and the control at room temperature of 25°C (CK). The physiological results suggested that leaves in both the 35°C and 40°C treatments showed maximum relative conductivity at 4 d of stress, increasing by 28.54% and 43.36%, respectively, compared to CK. Throughout the stress period (0–4 d), malondialdehyde (MDA) and soluble protein contents of raspberry leaves increased under HT35 and HT40 treatments, while soluble sugar content first decreased and then increased. Catalase (CAT) activity increased, superoxide dismutase (SOD) activity first increased and then decreased, and peroxidase (POD) activity gradually decreased. Photosynthetic and fluorescence responses of raspberry leaves showed the most severe impairment after 4 d of stress. Transcriptomics results revealed significant alterations in 42 HSP family genes, two SOD-related differentially expressed genes (DEGs), 25 POD-related DEGs, three CAT-related DEGs, and 38 photosynthesis-related DEGs under HTS. Kyoto Encyclopedia of Genes and Genomes (KEGG) analysis showed that these DEGs were mainly enriched in photosynthesis-antenna proteins, pentose and glucuronide interconversion, phenylpropane biosynthesis, and indole alkaloid biosynthesis. HTS induced excessive ROS accumulation in raspberry leaves, causing oxidative damage in plant cells and subsequently reducing photosynthesis in raspberry leaves. This reduction in photosynthesis, in turn, affects photosynthetic carbon fixation and starch and sucrose metabolism, which, combined with phenol propane biosynthesis, mitigates the HTS-induced damage.

## 1 Introduction

Temperature significantly affects plant growth and development, with normal plant growth occurring optimally within a certain temperature range. However, extreme heat stress (HS) weather is becoming more frequent, with high-temperature stress (HTS) disrupting intracellular environmental balance and severely inhibiting plant performance or even causing death (Kotak et al., 2007). Moreover, HTS causes curling, water loss, and sunburn of leaves (Szenteleki et al., 2012; Pablo et al., 2013), along with thickened pollen tubes, curled corollas, and reduced pollen viability (Hedhly, 2011). Moreover, high temperature induces early flowering, accelerated root growth, increased pathogen susceptibility, and thermal morphogenetic developments like reducing stomatal density and leaf thickness in plants (IbañEz et al., 2017; Zha, 2020). Furthermore, it shortens the flower life span, affects floral traits, reduces the nectar and pollen protein content, and impairs floral development and fertility, ultimately resulting in lower reproductive success (Descamps et al., 2021). Therefore, studying how HTS affects plants is imperative.

Raspberry (*Rubus idaeus* L.), a deciduous perennial shrub of the genus *Rubus*, family Rosaceae, is also known as pallet, mulberry, plucked berry, red berry, etc. They are eaten fresh, as well as processed into wine, juice, and jam (Zhang et al., 2020). Raspberry distribution spans temperate or Mediterranean climates in the western United States, southern Europe, Chile, Mexico, China, Japan, and Russia (Rambaran and Ginigini, 2020). Its fruits and leaves are rich in active substances like ellagic acid, superoxide dismutase (SOD), and flavonoids that have antioxidant, anti-tumor, anti-aging, and other effects, with diverse applications in medicine, cosmetics, etc. (God et al., 2010; Gevrenova et al., 2013). Additionally, recent studies have revealed that raspberry ketones in cranberries can reduce non-alcoholic fatty liver disease by regulating glycolipid metabolism and reducing oxidative stress. Furthermore, raspberry leaf extract showed antioxidant, antibacterial, and anti-inflammatory activities (Ma et al., 2022; Maslov et al., 2022). The high nutritional and medicinal value of raspberries opens possibilities for further development of therapeutics against lifestyle diseases gaining popularity worldwide, giving it the reputation of being the “fruit of life”.

Raspberries exhibit cold and drought resistance but are sensitive to high temperatures, making HS a significant limiting factor affecting raspberry production and cultivation (Swanson et al., 2011). Under HTS, plants undergo morphological changes and physiological and biochemical responses, including photosynthesis, respiration, cell membrane stability, sugar, lipid, and amino acid metabolism, as well as the expression of resistance genes and proteins. For example, cucumber responds to HTS by increasing its proline content, activating the antioxidant defense system, inducing stress protein production, and regulating phytohormone signaling pathways (Chen et al., 2021; Li et al., 2022). HTS causes dehydration in grape leaves and involves interactions between endogenous hormone-mediated defense processes in plant and abscisic acid (ABA) response pathways, with HSFA2 and HSFA7 being the most heat-sensitive (Zha, 2020). Transcription factors (ABR1, IAA26, OBF1, LUX, SCL3, DIV, NAC29, NAC72, and TCP3) may regulate the crosstalk between hormone signaling and HS response (HSR) in *Rhododendron* under HTS. Moreover, five raspberry varieties (‘Autumn ‘, ‘Britten’, ‘Autumn Bliss’, ‘Red Autumn’, ‘Korean Black’, and ‘Heritage’) showed altered amino acid and sugar metabolism, antioxidant metabolite accumulation, phytohormone-related MAPK signaling pathways, and α-linolenic acid metabolism (Zhu et al., 2022). Although studies on raspberries under HTS have mainly focused on physiological or molecular responses (combined transcriptional and metabolic analyses), no studies have focused on their combination. The results of the HSR studies differed between the different raspberry species.

Therefore, in this study, leaves of raspberry ‘Polka’ seedlings were treated at high temperatures (35°C and 40°C), with 25°C used as the control (CK). This was followed by studying how short-term moderate and severe HTS affects the physiological and molecular properties of red raspberry leaf cell membrane permeability, lipid peroxidation level, osmoregulation, antioxidant enzyme system, photosynthesis and chlorophyll fluorescence parameters, and transcriptome sequencing. Thus, the study results provide a more comprehensive insight into the raspberry HSR response, thus aiding in risk assessment and the development of essential measures to mitigate the adverse effects of heat on raspberry seedlings.

## 2 Materials and methods

### 2.1 Plant material, heat treatment

The HTS test was conducted on 22 August, 2020. Vigorously growing ‘Polka’ raspberry seedlings were selected under stable growing conditions, seedlings were ~11 cm high and 3 months old. They were then placed in a light incubator at 25°C with a 14-h photoperiod for 4 d. They were then subjected to HS at 35°C and 40°C, while 25°C served as a control for a 4-day treatment. Moisture was maintained during the stress treatment period. The experiment comprised three treatments with 10 plants per replicate and three replications, which totaled up to 90 plants. Each treatment was sampled at 0, 2, and 4 d post stress-treatment to determine their physiological indicators, and photosynthetic and chlorophyll fluorescence parameters. Transcriptome sequencing was performed on leaves at 0 and 4 d of stress treatment.

### 2.2 Determination of the physiological index of red raspberry (Polka) under HTS

Physiological indicators were measured in three replicates of red raspberry polka seedlings per treatment for each period. The chlorophyll content was determined by placing 2g of clipped leaves in 10 ml of acetone-ethanol solution until the leaves were completely discolored (Chen et al., 1989). Relative conductivity was measured with a conductometer (DDS-307A, INESAINSTRUMENT). The conductivity value for each treated leaf before boiling was recorded as *Ec1*. *Ec2* was the sample boiling water bath for two hours and then cooled to room temperature. Relative conductance=(*Ec1*/*Ec2*) × 100% (Li, 2000). Dried leaves weighing 0.1g were extracted three times in an 80 °C water bath with 80% ethanol. After merging the secondary extraction solution, a constant volume of 10ml was used as the soluble sugar test solution. Again, 0.1g dried leaves were extracted three times in an 80 °C water bath with 80% ethanol, and the secondary extraction solution was combined. The soluble sugar test solution was determined using anthrone colorimetry. Soluble protein from 2g of fresh leaves extracted with distilled water was stained with Coomassie Brilliant Blue G-250 staining (Divya et al., 2019). Antioxidant enzymes and malondialdehyde (MDA) were extracted from 0. 3 g of fresh leaves in a pH 7.8 phosphate buffered solution. The crude extract was assayed for SOD activity by using the nitrogen blue tetrazolium (NBT) photoreduction method, peroxidase (POD), and catalase (CAT) activity by using the active guaiacol and hydrogen peroxide methods, respectively (Jia et al., 2016). MDA content was assayed by thiobarbituric acid method (Li, 2000).

### 2.3 Determination of photosynthetic and chlorophyll fluorescence parameters of red raspberry (Polka) under HTS

The photosynthetic performance of raspberry ‘Polka’ of each treatment was assessed around 10:00 am on 0 d, 2 d, and 4 d of stress. Three replicates of functional leaves from raspberry ‘Polka’ tissue culture seedlings were chosen from each treatment, specifically from the 2^nd^–3^rd^ leaf position, for photosynthetic parameter determination. The net photosynthetic rate (*Pn*), transpiration rate (*Tr*), stomatal conductance (*Gs*), and intercellular CO_2_ concentration (*Ci*) were measured using a Li-6800 portable photo synthesizer (LI-COR of United States) equipped with a red and blue light source inside the leaf chamber. The light intensity was set at 800 μmol·m ^-2^·s^-1^ and the flow rate was at 500 μmol·s^-1^. The average values were subsequently calculated.

Chlorophyll fluorescence of the raspberry ‘Polka’ seedlings from each treatment was measured around 10:00 am on 0 d, 2 d, and 4 d of stress. Three functional leaves selected from the 2^nd^–3^rd^ leaf position were subjected to dark treatment for 20 min. Using a Pocket PEA fast fluorometer, the initial fluorescence (Fo), maximum fluorescence (Fm), and variable fluorescence (Fv) of the leaves were measured. The experimental data were analyzed using the IBM SPSS Statistics 27.0.1 software.

### 2.4 RNA extraction and sequencing

Three replicate test materials were selected from the same batch of red raspberry ‘Polka’ seedlings used for evaluating the physiological and biochemical indicators and were sent to Hangzhou Lianchuanr Biotechnology Ltd. (Hangzhou, China). Total RNA was extracted using the Tiangen RNA prep Pure Polysaccharide Polyphenol Plant Total RNA Extraction Kit. Then cDNA library construction and transcriptome sequencing (RNA- seq) were performed. After obtaining raw data, sequencing junctions were removed using cutadapted and unqualified sequences were filtered out using fqtrim to obtain clean data. eggNOG (http://eggnogdb.embl.de), GO, KEGG (www.genome.jp/kegg), Pfam (http://pfam.xfam.org/), NCBI-nr, Swiss-Prot, and other databases were used to annotate the genome functions.

Total RNA was extracted using PrimeScript™ 1st strand cDNA Synthesis Kit, (Takara, Japan). Quantitative reverse transcription PCR (qRT-PCR) was performed using the 2X SG Fast qPCR Master Mix (High Rox, B639273, BBI) according to the manufacturer’s protocol. The specific primers used for qRT-PCR were designed using Primer Premier 5.0 (**Table S1**). Each 20 μL qRT-PCR reaction contained 7.2 μL ddH_2_O, 0.4 μL of 10 μM primers F, and R, 10 μL of 2×SybrGreen qPCR Master Mix, and 2 μL of diluted 6 selected DEGs cDNA. The amplification program was: initiation by a 95°C for 3 min, followed by 45 cycles of 5s at 95°C, and 30s at 60°C, with a melting curve analysis program. Finally, the 96-well plate with the added samples was run on an ABI Step one plus fluorescent quantitative PCR instrument. Three technical replicates were performed for qRT-PCR.

## 3 Results

### 3.1 Physiological characteristics of red raspberry (Polka) under HTS

**Figure 1:**
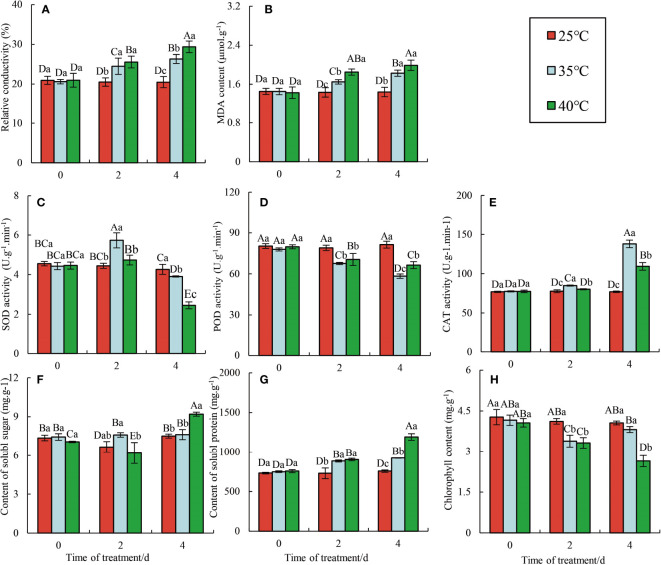
Effects of HTS on physiological and biochemical parameters in the Raspberry ‘Polka’ leaves. Data represent the means ± SE of three separate measurements. The significance of differences was analyzed using Duncan’s method. Lower case letters indicate the significance between the 0, 2, and 4 d treatments; upper case letters indicate the significance between all treatments within 4 d. **(A)** Changes in relative conductivity; **(B)** Changes in MDA content; **(C)** Changes in SOD activity; **(D)** Changes in POD activity; **(E)** Changes in CAT activity; **(F)** Changes in content of soluble sugar; **(G)** Changes in content of soluble protein; **(H)** Changes in chlorophy II content.

The normal physiological metabolism of plants is disrupted under HTS, resulting in external changes in plant leaves, including delayed height and diameter growth, wilting, color fading, and leaves dropping (Lehua et al., 2011; Divya et al., 2019). The plants were divided into three groups based on their HTS treatment: the 25°C group (CK), the 35°C group (HT35), and the 40°C group (HT40), with the last two being stressed for 2 d and 4 d, respectively. With the increasing stress time, raspberry plants showed different physiological characteristics under different high-temperature conditions (**Figure 1**). As expected, the physiological indexes of raspberry ‘Polka’ leaves remained relatively stable at 25°C. At 35°C, the chlorophyll content of raspberry ‘Polka’ leaves initially decreased and then increased, and gradually decreased again at 40°C (**Figure 1A**). Under HTS, the relative conductivity and MDA content of leaves increased (**Figures 1B, C**), indicating that high temperature severely damaged cell division and growth in raspberries (Zhao et al., 2018). Soluble sugar and soluble protein in leaves remained relatively stable, with only a gradual increase observed at 40°C (**Figures 1E, F**). In HSR, plants have evolved many protective scavenging or antioxidant defense mechanisms (Liu and Huang, 2000). The SOD and CAT activities in leaves treated with 35°C were always higher than those treated with 40°C, whereas the POD activity in leaves treated with 40°C showed the complete opposite (**Figure 1D**). Overall, there were significant differences in the effects of each treatment at 4 d. However, this protection mechanism failed beyond its threshold, with different antioxidant enzymes having different thresholds. The changes in physiological indexes also suggest the involvement of complex molecular mechanisms in the HSR of ‘Polka’ raspberry.

### 3.2 Photosynthesis and chlorophyll fluorescence of red raspberry (Polka) under HTS

**Figure 2:**
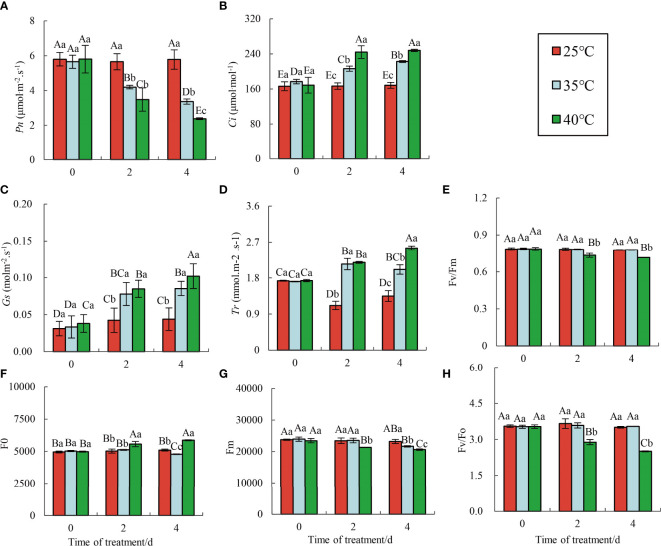
Changes in chlorophyll fluorescence parameters under HTS. Data represent the means ± SE of three separate measurements. The significance of differences was analyzed using Duncan’s method. Lower case letters indicate the significance between the 0, 2, and 4 d treatments; upper case letters indicate the significance between all treatments within 4 d. **(A)** Changes in Pn; **(B)** Changes in Ci ; **(C)** Changes in Gs; **(D)** Changes in Tr; **(E)** Changes in Fv/Fm; **(F)** Changes in Fo; **(G)** Changes in Fm; **(H)** Changes in Fv/Fo.

The change of *Ci* is the main determinant of the photosynthetic rate. Chlorophyll a fluorescence determination can quickly, accurately, and effectively evaluate the plant photosynthetic response. With increasing HTS time, the *Pn* of red raspberry ‘Polka’ showed a significant downward trend (**Figure 2A**). Moreover, *Pn* was significantly lower at 40°C compared to 35°C. Conversely, *Ci* (**Figure 2B**), *Gs* (**Figure 2C**), and *Tr* (**Figure 2D**) increased significantly under different high-temperature conditions, peaking at 4 d of stress, with the highest value observed at 40°C.

Generally, HSR and domestication mechanisms enable plants to adapt to new environments and maintain photosynthesis (Horton et al., 2008). However, when stress exceeds the adaptive capacity, perpetual photo-inhibition occurs. Strong, severe, or prolonged stress decreases the useful quantum yield and photochemical quenching (q P) of PSII (φ PSII), thus inhibiting the electron transport chain. Severe stress conditions may lead to a significant loss of PSII function (Nabity et al., 2013). The fluorescence parameters of raspberry ‘Polka’ remained relatively stable at 25°C, while the maximum photochemical efficiency (Fv/Fm) (**Figure 2E**) and potential activity of PSII (Fv/Fo) (**Figure 2H**) exhibited different changes under different HTS. Fv/Fm and Fv/Fo of leaves treated at 35°C showed no significant difference from CK but gradually decreased at 40°C. As stress prolonged, Fo decreased slowly at 35°C and increased gradually at 40°C (**Figure 2F**). Additionally, Fm decreased gradually in all high-temperature treatments (**Figure 2G**), consistent with previous findings on *Pinellia Ternate* (Xue et al., 2010).

### 3.3 Transcriptome sequencing and quality assessment

The molecular mechanism of high-temperature resistance of raspberry ‘Polka’ was evaluated using the Illumina Hiseq platform based on synthetic sequencing (SBS) technology. The cDNA libraries of each treatment were sequenced, and the results showed high validity (94.9–97.8%), with Q20 > 98%, Q30 > 96%, and GC content > 46%, indicating good sequencing quality and could thus be used for subsequent analysis. The *de novo* assembled valid reads were clustered to remove redundancy, resulting in 15,596 Unigenes (32.18%) and 13,191 Unigene (27.21%) distributed within 200–300 nt and 300–700 nt (**Table S2**). The assembly demonstrated good integrity, good sequencing effect, and high reliability for further research purposes.

### 3.4 Analysis of differentially expressed genes (DEGs)

**Figure 3:**
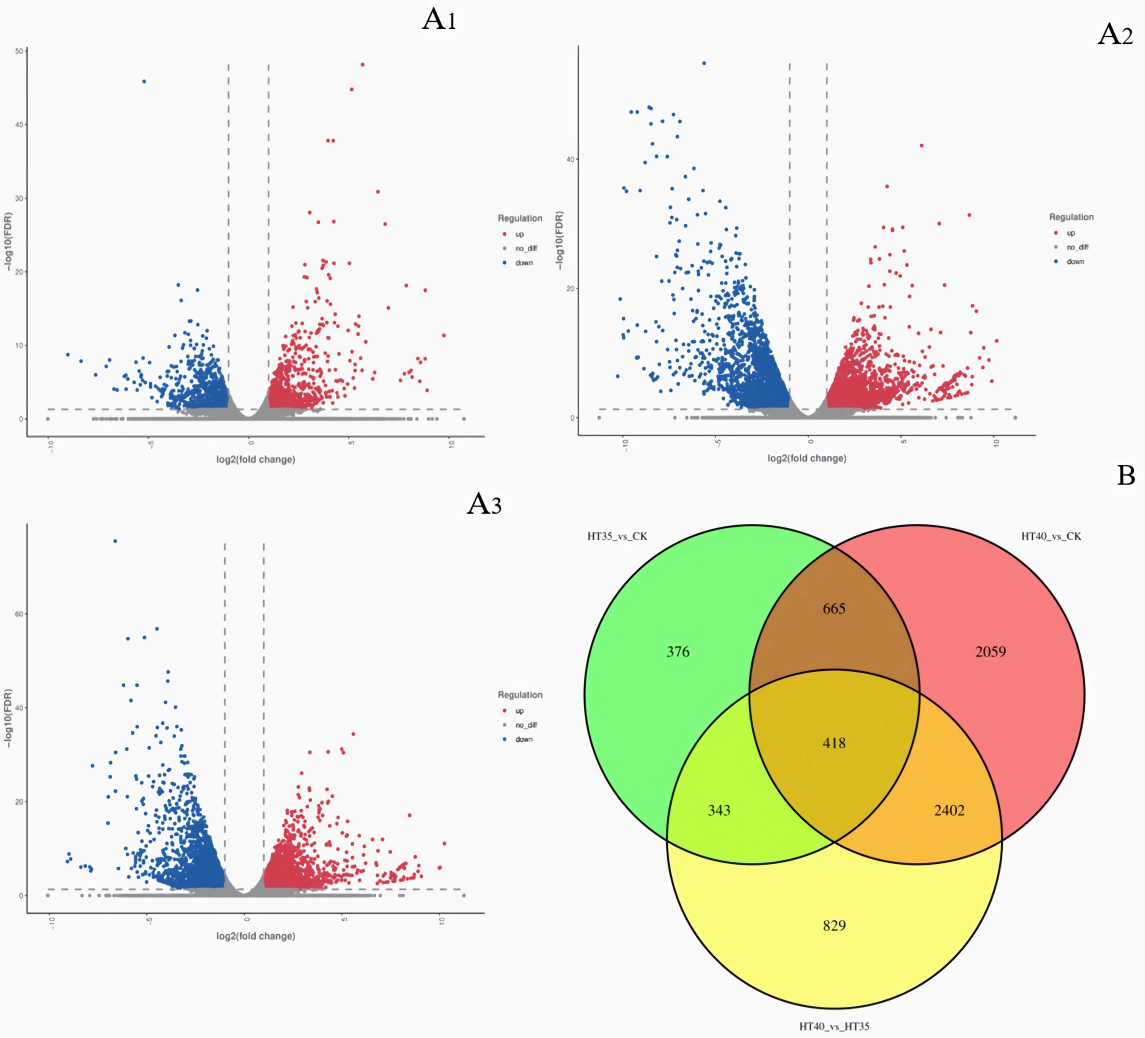
Heat map of DEGs under HTS. **(A1)** DEGs volcano map of HT35_vs_CK; **(A2)** DEGs volcano map of HT40_vs_CK; **(A3)** DEGs volcano map of HT40_vs_HT35; **(B)** Venn diagram of all differential genes. The CK, HT35, and HT40 refer to the differences between the mean values of three biological replicates of samples treated at 25°C, 35°C, and 40°C for 4 days, respectively.

**Figure 3** showed that HT35_vs_CK had a total of 1082 DEGs, with 920 upregulated and 882 downregulated (**Figure 3A1**). HT40_vs_CK had a total of 5544 DEGs, with 2872 upregulated and 2672 downregulated (**Figure 3A2**). HT40_vs_HT35 had 1823 and 2169 upregulated and downregulated DEGs, respectively (**Figure 3A3**). Transcriptome data analysis revealed 1,426 DEGs common between HT35_vs_CK and HT40_vs_CK. Additionally, there were 761 DEGs common between HT35_vs_CK and HT40_vs_HT35, while 2,820 DEGs between HT40_vs_CK and HT40_vs_HT35. A total of 418 DEGs were identified between treatment groups (**Figure 3B**).

### 3.5 Gene Ontology (GO) analysis

**Figure 4:**
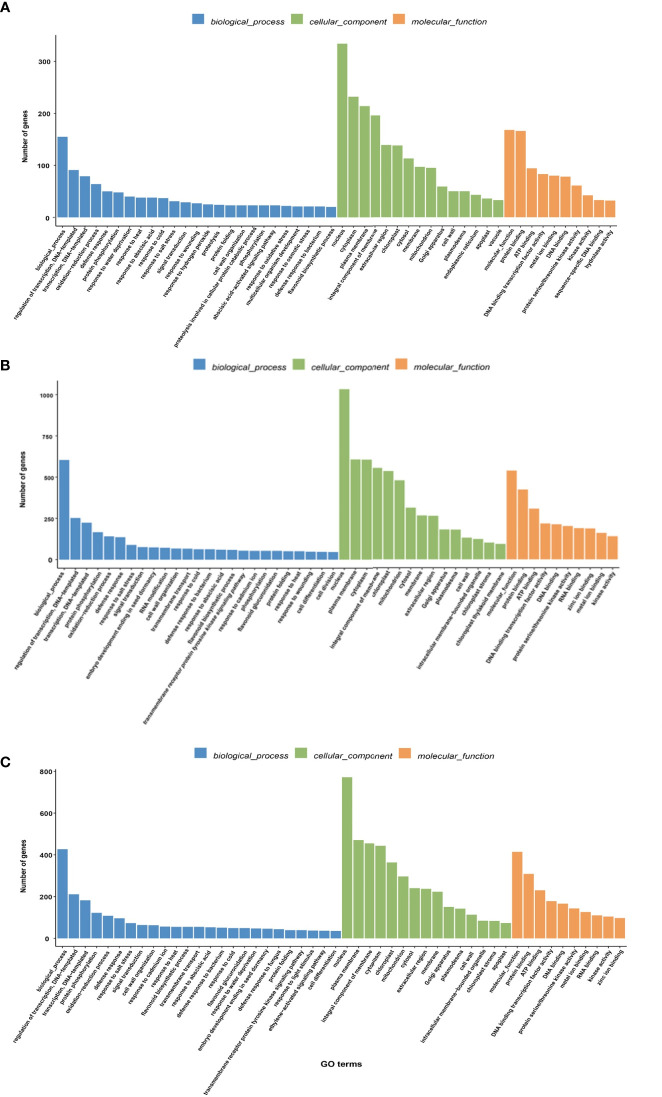
GO enrichment analysis of DEGs. The abscissa represents the ratio of the genes annotated to the entry to the total number of genes annotated, while the ordinate represents the name of the GO entry. The label at the top of the Figure represents the category containing the GO entry. **(A)** DEGs heat map of HT35_vs_CK; **(B)** DEGs heat map of HT40_vs_CK; **(C)** DEGs heat map of HT40_vs_HT35.

The GO notes had three functional categories including “molecular function,” “cellular component,” and “biological processes” (**Figure 4**). DEGs showed significant enrichment for GO terms in the GO database. Regarding cellular components, the three combinations of HT35_vs_CK, HT40_vs_CK, and HT40_vs_HT35 exhibited similar significant GO enrichment terms, including the plasma membrane, chloroplast, cytoplasm, membrane, mitochondria, Golgi apparatus, cell wall, chloroplast thylakoid membranes, etc. (**Figure 4**).

In terms of biological processes, the combinations HT35_vs_CK and HT40_vs_CK demonstrated defense responses, responses to heat, flavonoid biosynthesis processes, responses to abscisic acid, and oxidized lipid biosynthesis processes. HT35_vs_CK specifically showed enrichment in response, oxidative stress response, and response to osmotic stress, while HT40_vs_CK displayed enrichment in photoinhibition, redox process, and photosynthesis (**Figures 4A, B**).

Furthermore, in the HT40_vs_HT35 combination, there was significant enrichment in chlorophyll biosynthesis process, photosynthesis and photosynthesis in photosystem I, light harvesting, etc. (**Figure 4C**). The HT40_vs_HT35 combination also exhibited significant enrichment in chlorophyll biosynthesis process, photosynthesis and photosynthesis in photosystem I, light harvesting, etc.

### 3.6 Kyoto Encyclopedia of Genes and Genomes (KEGG) pathways enrichment analysis under HTS

**Figure 5:**
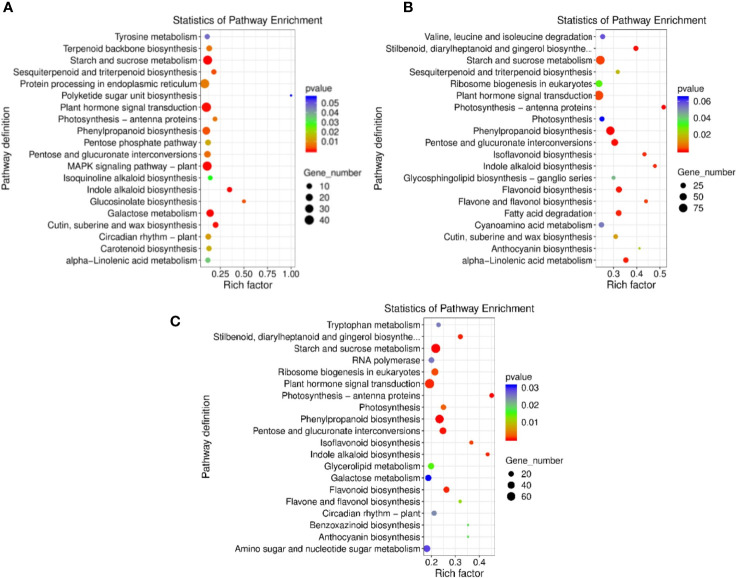
KEGG enrichment analysis of DEGs. KEGG enrichment is measured by rich factor, q-value, and the number of genes enriched in this pathway. **(A)** DEGs heat map of HT35_vs_CK; **(B)** DEGs heat map of HT40_vs_CK; **(C)** DEGs heat map of HT40_vs_HT35.

The DEGs obtained by comparing the double-season red raspberry ‘Polka’ under different high-temperature treatments were searched in the KEGG database to obtain its metabolic pathway entries. The HT35_vs_CK, HT40_vs_CK, and HT40_vs_HT35 combinations had 798, 2367, and 1805 genes (**Figure 5**) and 18, 18, and 23 pathways, respectively. The top 20 KEGG enrichment pathways of DEGs were plotted as scatter plots according to enrichment factors (**Figure 5**). In HT35_vs_CK, the most enriched pathway was the biosynthesis of polyketone units, followed by biosynthesis of glucosinolates. In HT40_vs_CK and HT35_vs_HT40, the most enriched degree was photosynthesis-antenna protein, followed by indole alkaloid biosynthesis (**Figures 5A, B**). Indole alkaloid biosynthesis, pentose and glucuronic acid interconversion, phenylpropanoid biosynthesis, and photosynthesis-antenna were among the top 20 enriched pathways in all three comparison groups.

### 3.7 Differentially expressed genes of HSPs

**Figure 6:**
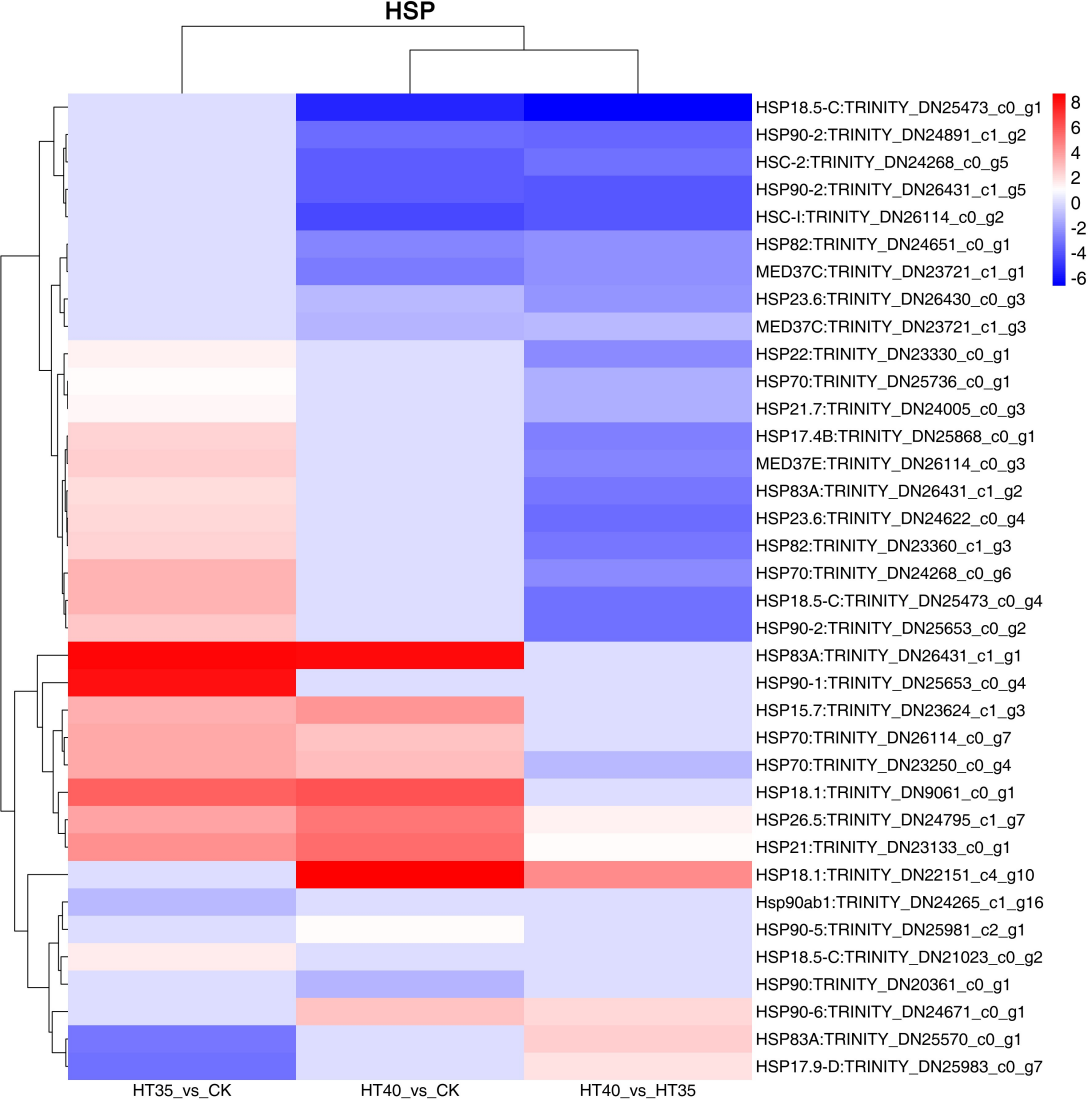
DEGs related to HSPs. The numbers on the different color bars are the log2 converted values.

The significant differential expression of HSP family genes was observed under HTS (**Figure 6**), with 36 DEGs. In HT35_vs_CK, 20 and three genes were upregulated and downregulated, respectively. The highest fold upregulation was observed in HSP83A: *TRINITY_DN26431_c1_g1*. In HT40_vs_CK, 10 genes were upregulated and 10 genes were downregulated, where HSP18.1: *TRINITY_DN22151_c4_g10* was upregulated and HSP18.5-C: *TRINITY_DN25473_c0_g1* was downregulated by the highest fold. In the HT35_vs_40, six genes were upregulated and 21 genes were downregulated, where HSP18.5-C: *TRINITY_DN25473_c0_g1* was downregulated by the highest fold. Further analysis of raspberry HTSDEGs HSP family members revealed the presence of eight HSP90 homologs, five HSP18 homologs, four HSP70 homologs, and three HSP83 and MED37 homologs. This indicated the complexity of HSP90, HSP18, and HSP70 homologs, with HSP18 showing more fluctuations in up- and downregulated ploidy across the three treatment groups. These findings suggest that these homologs may be crucial in DEGs HSR in raspberries. Both molecular chaperones, HSP70 and HSP90, played important roles in protein folding, assembly, and transfer under HTS, while HSP18 may improve tolerance and resistance to high temperatures. Previous studies in plants such as chrysanthemum (Shi et al., 2022) and grape (Zha, 2020) have also reported the involvement of HSP70 and HSP18 in heat tolerance.

### 3.8 Analysis of DEGs related to physiological indicators under HTS

**Figure 7:**
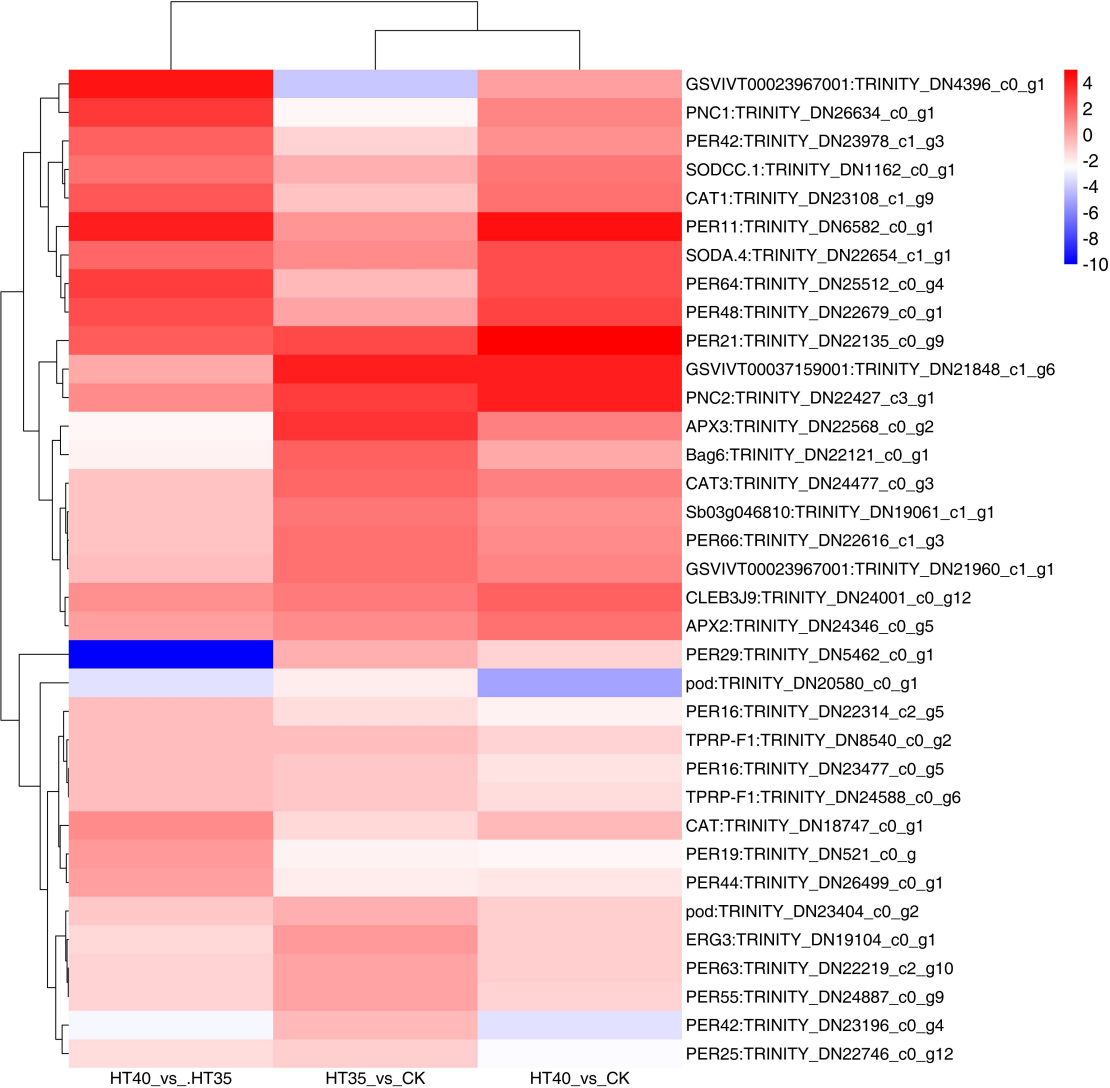
DEGs related to physiological indicators under HTS. The numbers on the different color bars are the log2 converted values.

DEGs related to antioxidant enzymes and osmoregulatory were screened (**Figure 7**). There were three DEGs about ascorbate peroxidase that were upregulated under both HT35 and HT40, namely APX3: *TRINITY_DN22568_c0_g2*, CLEB3J9: *TRINITY_DN24001_c0_g12* and APX2: *TRINITY_DN24346_c0_g5*. There were two DEGs associated with SOD, both of which were upregulated in HT40_vs_CK and HT40_vs_HT35, namely SODA.4: *TRINITY_DN22654_c1_g1* and SODCC.1: *TRINITY_DN1162_c0_g1*. It had 23 POD-related DEGs, where 10 and 13 genes were upregulated and downregulated in HT35_vs_CK and HT40_vs_HT35, respectively. Furthermore, eight genes were upregulated and 11 genes were downregulated under HT40_vs_CK. There were three CAT-related DEGs, among which CAT1: *TRINITY_DN23108_c1_g9*, CAT: *TRINITY_DN18747_c0_g1* and CAT3: *TRINITY_DN24477_c0_g3*. And CAT1: *TRINITY_DN23108_c1_g9* and CAT: *TRINITY_DN18747_c0_g1* were downregulated, while CAT3: *TRINITY_DN24477_c0_g3* was upregulated in HT35_vs_CK. However, the opposite was seen in HT40_vs_HT35. There were four DEGs associated with proline, where TPRP-F1: *TRINITY_DN24588_c0_g6*, ERG3: *TRINITY_DN19104_c0_g1*, TPRP-F1: *TRINITY_DN8540_c0_g2*, and Bag6: *TRINITY_DN22121_c0_g1*. Generally, the more the genes were enriched, the more HTS-responsive they were. In summary, POD and proline enriched 23 and four genes, respectively, indicating that they may enhance the HTS tolerance of raspberry. HTS induces the production and accumulation of reactive oxygen species (ROS) in plants, thereby enhancing antioxidant enzyme activity. The increase in antioxidant enzyme activity promotes the elimination of different types of HTS-generated ROS [singlet oxygen (^1^O_2_), superoxide (O^−2^), hydrogen peroxide (H_2_O_2_), and hydroxyl radical (OH^−^)] (Medina et al., 2021). The increase in proline content promotes osmoregulation and maintains normal light cooperation to improve plant heat tolerance (Zouari et al., 2016). Additionally, excessive ROS accumulation produces abundant NO to induce CaM3 expression and promote the expression of related HSP genes (Zhu et al., 2021). Our transcriptome data mainly analyzes HSP expression and ROS production and evaluated genes and enzymes related to these two pathways that showed transcriptional regulation in raspberries. However, the precise response mechanism of these pathways to HS is currently unclear.

### 3.9 Photosynthesis synthesis and response-related genes

**Figure 8:**
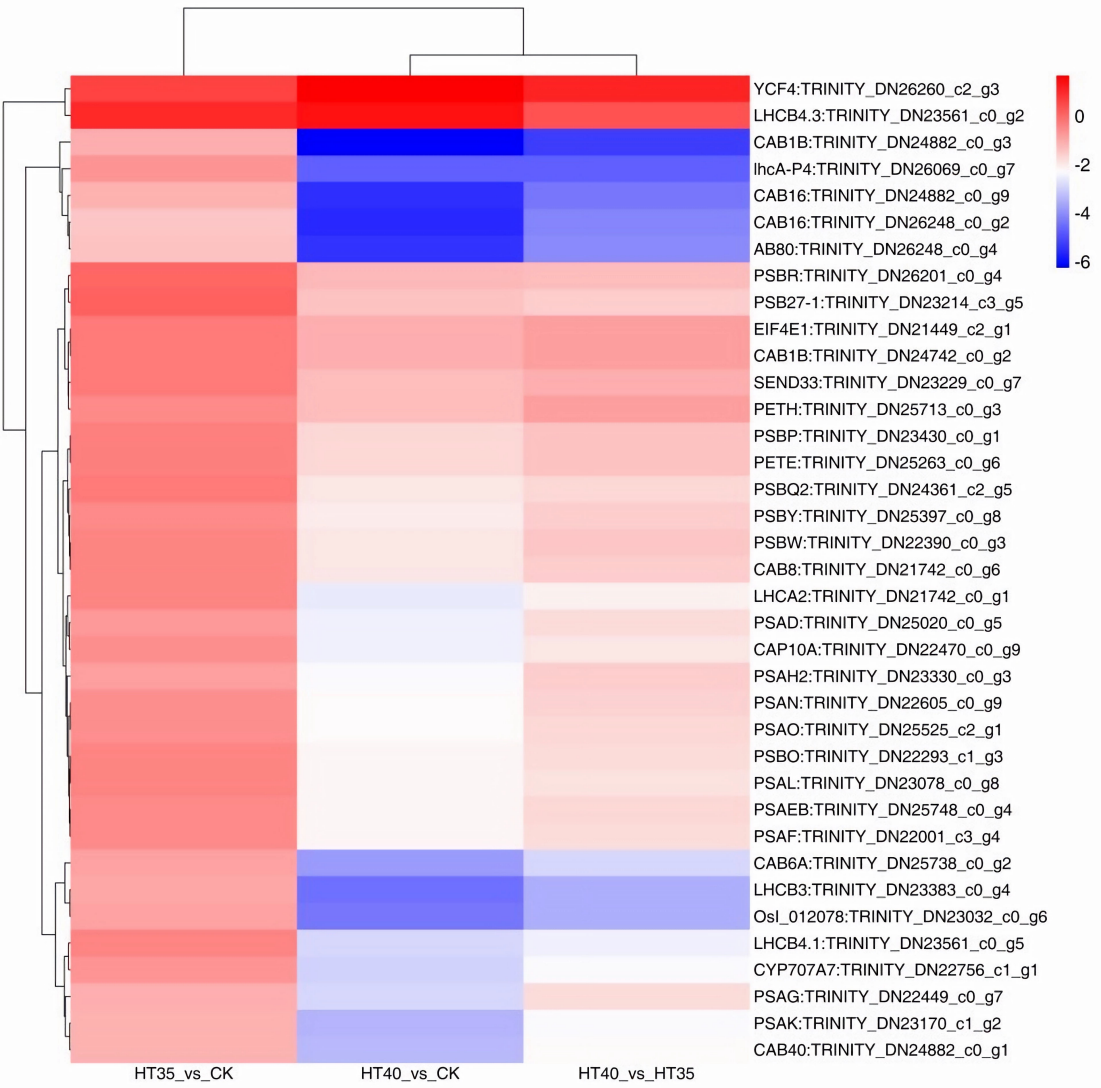
DEGs related to the photosynthetic under HTS. The numbers on the different color bars are the log2 converted values.

Photosynthesis is an essential process for plant growth, and chlorophyll a fluorescence shows temperature sensitivity. The result shows that the number of photosynthetic response genes differentially expressed between HT40_vs_HT35, HT40_vs_CK was higher than HT35_vs_CK (**Figure 8**). Among the 38 DEGs obtained under HTS, HT35 had one DEG and eight lowered gene expressions. Contrastingly, HT40 had 36 DEGS, of which two were upregulated and 35 were downregulated (**Figure 8**). In the optical-electronic chain, the expressions of three genes that encoded the blue in, iron restore protein, and iron reduction protein NADP^+^ reduction enzyme were all reduced, specifically *TRINITY_DN25263_c0_g6*, *TRINITY_DN23229_c0_g7*, and *TRINITY_DN25713_c0_g*3. Additionally, cell pigmentation, B6F complex (CYTB6F), PSI, and PSII were injured. Among the genes encoding CYTB6F, one gene was downregulated, with nine and seven genes encoding PSI and PSII, respectively, also being downregulated.

Under HTS, the antenna chlorophyll A/B protein complex (LHC) of PSI and PSII, also known as the optical protein composite, was damaged. At 35°C, there were six DEGs encoding LHCII in the PS II, with five downregulated and one upregulated. At 40°C, the differential expression of LHCI coding genes was observed in photosystem I, with all four genes being downregulated. In PS II, there were 11 DEGs encoding LHCII, with one gene upregulated and 10 genes downregulated. In *Haematococcus pluvialis* subjected to abiotic stress, genes associated with photosynthetic antennae proteins such as *LHCA1*, *LHCB2*, *LHCB3*, and *LHCB7* were partially upregulated (He et al., 2018).

### 3.10 Changes in related pathways in red raspberry (Polka) under HTS

**Figure 9:**
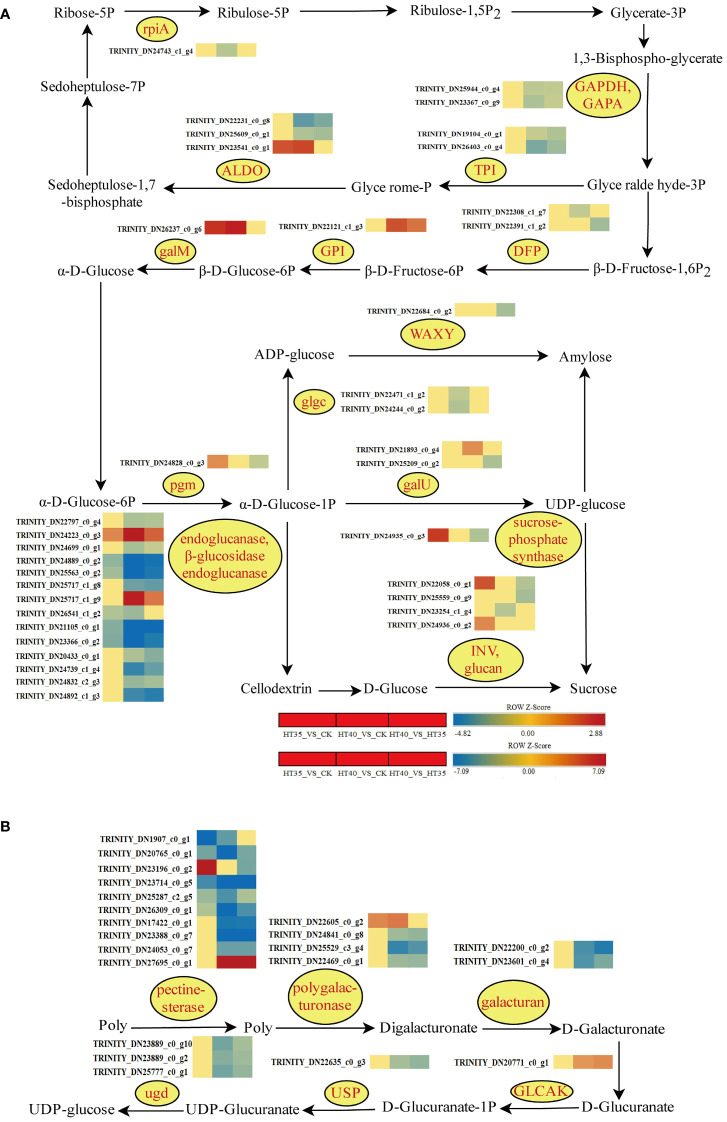
Photosynthetic carbon fixation and synthesis of sucrose and starch, as well as the transformation between pectin and glucose. Gene changes under HTS. The color block represents the log2FC value. Red and blue indicate significant upward and downward corrections. **(A)** Heat map of HSR of DEGs involved in photosynthetic carbon fixation, sucrose, and starch synthesis pathways under HTS. **(B)** Heat map of HSR DEG of the transformation pathway between pectin and glucose under HTS.

Genetic changes in photosynthetic carbon fixation and sucrose/starch synthesis pathways were examined to investigate the pathways associated with HTS. In the photosynthetic carbon sequestration pathway, the expression level of HT35 decreased, while controlling the synthesis of GAPDH, GAPA, and TPI genes for G-3P and G-P showed a decrease at HT40_VS_CK and HT40_VS_HT35 (**Figure 9A**). In the sugar and starch synthesis pathway, the β- DFP gene for D-Fructose-6P synthesis exhibited downregulation in HT40_VS_CK and HT40_VS_HT35 treatment groups. Conversely, the expression of the GPI and galM genes, involved in β-D-Glucose-6P and α-D-Glucose synthesis, respectively, increased under HTS (**Figure 9A**). Amylose synthesis in red raspberry leaves occurs mainly through two pathways: one involves ADP-glucose production from α-D-Glucose-1P, catalyzed by glgcase, followed by amylose production via the WAXY enzyme. The other pathway involves amylose synthesis from α-D-Glucose-1P with galU-producing UDP-glucose. Glucose synthesis involves the conversion of α-D-Glucose-1P into UDP-glucose in the presence of galU and sucrose through the action of sucrose phosphate synthase, followed by the INV and glucan enzymes to produce D-Glucose. Another pathway was the synthesis of D-Glucose from cellodextrin via the endoglucanase and β-glucosidase endoglucanase enzymes (**Figure 9A**). The expressions of glgc (responsible for ADP-glucose synthesis) and WAXY (involved in amylose synthesis) decreased under HTS. The endoglucanase and β-glucosidase endoglucanase genes controlling cellodextrin synthesis were downregulated to varying degrees, except for *TRINITY_DN24223_c0_g3* and *TRINITY_DN25717_c1_g9*, with both being upregulated (**Figure 9A**). Different heat treatments resulted in different gene expression patterns, indicating different effects on sucrose and starch synthesis in red raspberry. HT40 treatment showed higher expression of cellulose into glucose compared to HT35, indicating higher metabolic breakdown of sucrose into glucose and enhanced glucose synthesis. In the pectin-glucose conversion pathway, the expression of the pectinesterase, polygalacturonase, and galacturan genes involved in D-Galacturonate synthesis was downregulated at different high-temperature levels, particularly in HT40 as compared to HT35. However, the expression of ugdase genes responsible for UDP-glucose synthesis was reduced (**Figure 9B**).

**Figure 10:**
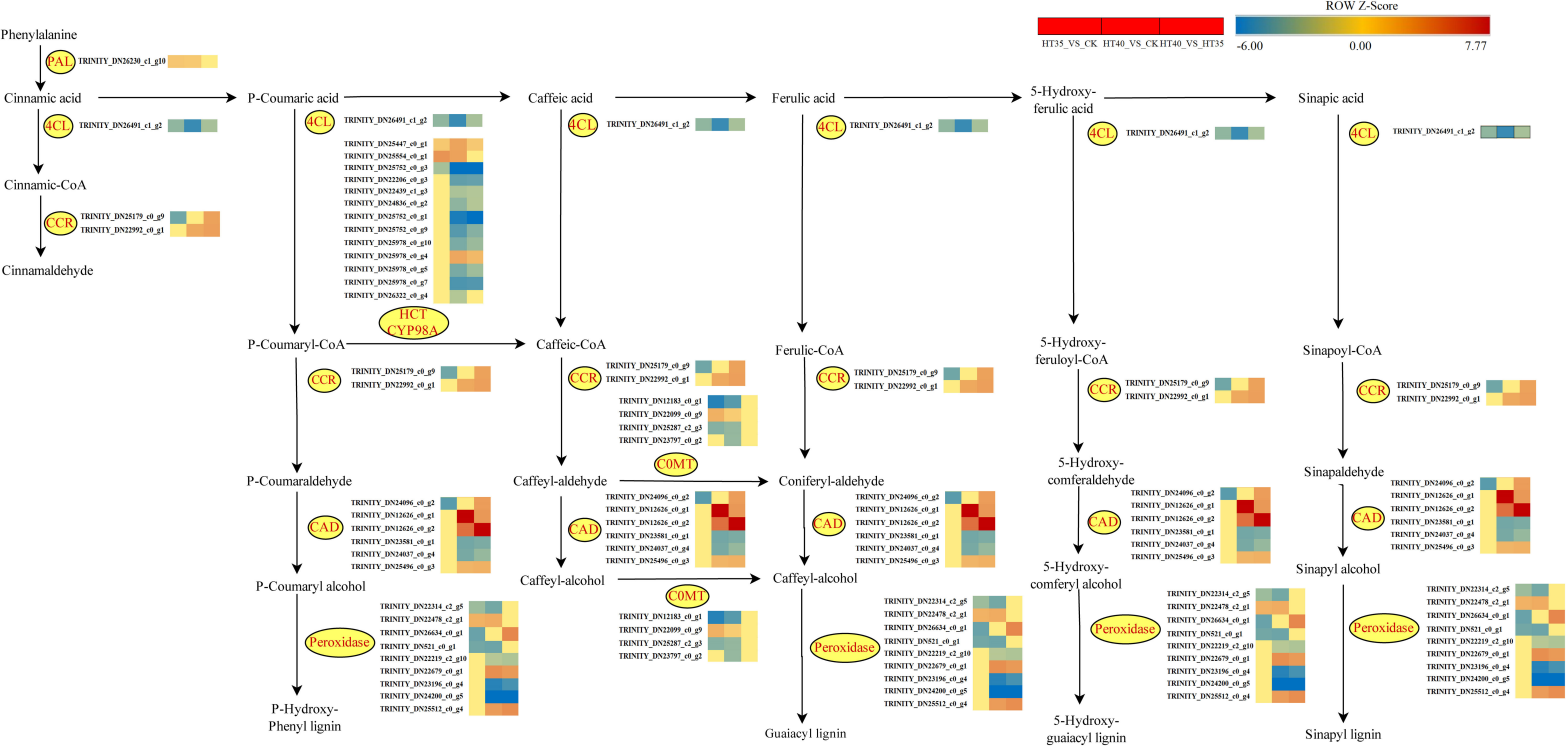
Changes in phenylpropanoid biosynthesis genes under HTS. The color block represents the log2FC value. Red and blue indicate significant upward and downward gene correction.

Phenylpropane biosynthesis plays a vital role in non-stress biological aspects. Phenylalanine is converted into trans-cinnamic acid by PAL (phenylalanine ammonia-lyase), which further reduces cinnamaldehyde through the actions of 4CL and CCR (**Figure 10**). Additionally, p-Coumarin acid, caffeic acid, ferulic acid, 5-Hydroxyferulic acid, and sinapic acid generated p-Hydroxyphenyl lignin, Guaiacyl lignin, 5-Hydroxyguaiacyl lignin, and Sinapyl lignin, respectively under the actions of 4CL, CCR, CAD, and POD. Notably, in the p-hydroxyphenyl lignin pathway, p-Coumaryl-CoA is converted to caffeic-CoA through HCT and CYP98A, while caffeoyl-aldehyde and caffeoyl-alcohol are converted into coniferyl-aldehyde and caffeoyl-alcohol, respectively, via COMT, ultimately leading to guaiacol lignin. The PAL-encoding gene was upregulated in the HT35_VS_CK and HT40_VS_CK treatment groups, while the expression of gene synthesizing COMT varied significantly, but did not show significant changes in HT40_VS_HT35, indicating similar levels of gene upregulation under different stress conditions. Among the genes controlling CAD synthesis, there were no significant changes observed except for *TRINITY_DN24096_c0_g2*, which was downregulated in HT35 treatment (**Figure 10**). Other genes showed significant up- and down-regulation trends in HT40_VS_CK HT40_VS_HT35 treatment groups. The gene regulating the synthesis of CCR was downregulated by HT35 and upregulated by HT40 treatment. The expression involved in POD, HCT, and CYP98A synthesis increased in the HT40_VS_CK, HT40_VS_HT35 treatment groups, while the genes controlling the synthesis of these two species showed decreased expression under mild HS. These gene changes indicated an increase in lignin and cinnamaldehyde synthesis in red raspberry leaves under HTS, with their accumulation being related to the stress intensity. However, the gene expression of their synthesis-related genes remains unchanged after reaching the stress threshold.

### 3.11 qRT-PCR validation of differentially expressed transcripts from RNA-Seq

**Figure 11:**
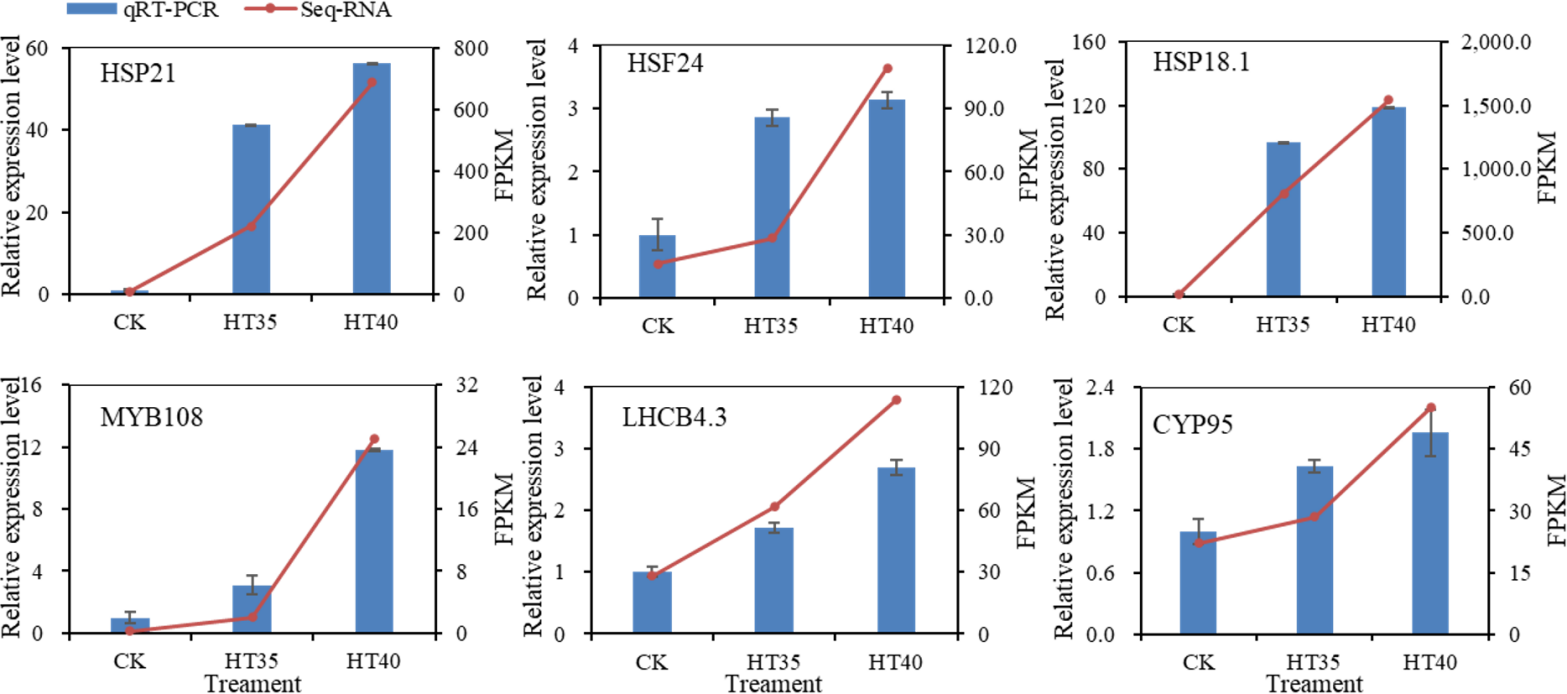
The expression patterns of six DEGs screened from HT35 and HT40 were obtained using RNA-seq and qRT-PCR. Different treatments (x-axis) and gene expression (y-axis) are shown by Fragments Per Kilobase of transcript per Million mapped reads (FPKM) (right) and relative expression levels log2FC (left). The blue bar in all plots is the relative expression obtained by qRT-PCR; the red line indicates the FPKM value obtained by RNA-seq. Bars indicate standard errors (n=3).

qRT-PCR detected the expression levels of six key genes under HTS. **Figure 11** shows significant changes (*p*<0.05) in the expression levels of all six genes after high-temperature treatment in red raspberry. The gene expression validation results were generally consistent with the gene expression trends determined by transcriptomic results, thus confirming the confidence and precision of this study’s RNA-Seq analysis.

## 4 Discussion

### 4.1 Physiological and biochemical differences HTS

HS exerts diverse effects on physiological, biochemical, and developmental processes in plants, including photosynthesis, respiration, membrane stability, and enzyme activity, ultimately affecting yield and quality. In this study, the chlorophyll content of red raspberry ‘Polka’ leaves initially decreased and then increased with increasing stress duration at 35°C (**Figure 1A**). Interestingly, chlorophyll synthesis can persist in high-temperature environments, providing protection against photo-oxidation for *S. viminalis* (Mlinaric et al., 2016). However, at 40°C, chlorophyll content continued to decrease, possibly due to a reduced synthesis rate or an increase in decomposition (Djanaguiraman et al., 2014). The simultaneous occurrence of chlorophyll fluorescence and PS II-mediated energy transfer process of converting light energy into stable chemical energy is considered a prominent indicator of HTS expression (Anjana et al., 2014). Meanwhile, red raspberry leaves showed significant changes in photosynthesis and fluorescence responses. As stress duration and intensity increased, the *Pn* in the red raspberry ‘Polka’ leaves gradually decreased, while *Ci*, *Tr*, and *Gs* gradually increased. Additionally, Fv/Fm, Fv/Fo, and Fm decreased, while Fo increased (**Figure 2**). HTS suppressed *Pn* increase and *Gs* decrease, while *Ci* stabilized and Rubisco activity decreased (Zhai et al., 2020). During photosynthesis, a decrease in *Gs* leads to reduced *Tr*, which subsequently significantly reduced transpiration, thereby inhibiting water and nutrient uptake and transport (Gao et al., 2016). Conversely, stress conditions reduced the chlorophyll fluorescence parameter Fv/Fm, indicating high temperature-induced inhibition of photosynthetic responses due to the instability of the cystoid structure and electron flow in the photosystem (Li et al., 2015). Furthermore, the relative conductivity, MDA content, and soluble protein content in red raspberry ‘Polka’ leaves increased, while soluble sugar content first decreased and then increased. CAT activity increased, SOD activity first increased and then decreased, and POD activity gradually decreased with the increasing stress duration (2 d to 4 d) and elevated stress levels from 35°C to 40°C (**Figure 2**). These results suggest that raspberry’s protective mechanisms may be triggered only during a sufficient exposure time to HTS, including ROS scavenging by antioxidant enzymes. Notably, HTS affects the physiological and biochemical indexes of many plants (Jia et al., 2016; Lee et al., 2018). These research results are consistent with those of *Rhododendron hainanense* (Zhao et al., 2018), grapes (Zha, 2020), and potatoes (Tang et al., 2020). Higher temperatures improve respiration and promote sugar consumption, while soluble sugars act as osmoregulation to maintain cell expansion through the concentration gradient (Ghanem et al., 2009).

### 4.2 Mechanisms regulating photosynthetic carbon fixation, sucrose starch synthesis, and pectin glucose conversion under HTS

Photosynthesis is the foundation for plant growth and development, providing essential substances and energy. Previous studies have shown that HTS significantly alters plant photosynthesis, with sustained intense heat causing changes in raspberry physiological and biochemical indicators, including chlorophyll degradation, reduced photosynthetic activity, and increased osmoregulation. Similar results are shown in **Figures 1**, **2**. Raspberries, being one of the best-selling and in-demand small berries, are characterized by early fruiting, a long fruiting period, high yields, and high profits. The raspberry ‘Polka’ had both summer and autumn fruiting seasons, with high temperatures being one of the major constraints. Photosynthesis is a key step in converting light energy to ATP, NADPH, and CO_2_ into carbohydrates. Under HTS conditions, genes involved in photosynthetic carbon fixation undergo changes, resulting in marked genetic variations in the synthesis of sugar and starch synthesis. ATP and NADP during photosynthetic carbon sequestration serve as precursors for carbohydrate biosynthesis. Additionally, GAPA and PGK enzymes consume ATP and NADP to convert glycerol-3-phosphate to glyceraldehyde-3-phosphate, a crucial Calvin cycle component (Zhang et al., 2021). Under HT40 treatment, phosphorylated glyceraldehyde-3-phosphate dehydrogenase (NADP^+^) (GAPA), phosphotrisaccharide isomerase (TPI), fructose bisphosphate aldolase class I (ALDO) enzyme and ribose 5-phosphate isomerase (rpiA) decreased, while the HT35 treatment showed no significant change (**Figure 9A**). Additionally, the down-regulation of GAPA and GAPB expression levels caused changes in soluble sugar accumulation and energy (Gd et al., 2022). This suggests that HT40 treatment may reduce soluble sugars in the chloroplast and inhibit the supply of 1,3-diphosphoglycerate during glycolysis, thus reducing the photosynthetic carbon sequestration capacity.

Osmoregulations (soluble sugars, starch, and soluble proteins) are important for regulating the internal plant cell pressure and maintaining the plant’s physiological stability. Osmoregulations are found to accumulate with increasing external abiotic stress, and starch can be hydrated to glucose in addition to its role in photosynthesis (Gudesblat et al., 2009). Endoglucanase and b-glucosidase are integral enzymes for cellulose breakdown into glucose sugars. The export of enzymes involved in the cellulose breakdown is reduced under HTS, resulting in an increase in cellulose content and a decrease in glucose content. The expression of genes controlling endoglucanase and B-glucosidase is downregulated under HTS (**Figure 9A**), indicating reduced cellulose breakdown to glucose less, which was consistent with the reduction in soluble sugar content under 2d HT40 treatment (**Figure 1F**). The reduction in leaf starch content under HTS may promote the conversion of starch to soluble sugars, which in turn promoted sucrose accumulation.

Studies related to the conversion of pectin to glucose are limited. Pectin mainly affects secondary wall thickening in fibrous and woody structures and is generally biosynthesized *via* the D-galacturonate, myo-inositol, and UDP-glucose pathways. The ugdase gene, which controls UDP-glucose synthesis, is downregulated in red raspberries under HTS (**Figure 9B**), similar to the sugar-starch synthesis pathway.

### 4.3 Regulation mechanism of lignin biosynthesis under HTS

Under HTS, plants experience ROS disruption, causing excessive ROS accumulation and resulting in plant cellular structural damage, reduced physiological functions, and retarded growth and development (Zhao et al., 2018). Therefore, plants can mitigate the HTS-induced damage through a series of physiological and biochemical reactions, including affecting chlorophyll biosynthesis, promoting stomatal closure, inhibiting photosynthesis, and evolving antioxidant enzyme systems like SOD, CAT, and POD (Zha, 2020). Antioxidant enzymes can effectively remove the excessive accumulation (**Figure 1D**) with the CAT content increasing (**Figure 1E**). In contrast, SOD content in HT35 first increased and then decreased, and it kept decreasing in HT40 (**Figure 1C**). Lignin is vital for sustaining the plant vitelline tissue t (Liu et al., 2018). In particular, the hydrophobic properties of lignin make the cell structure impermeable to water, which facilitates the maintenance of normal expansion of plants under HTS. POD and lactase (LAC) polymerize lignin in the secondaries with three main monophenols, thus increasing the plant’s phenolic and lignin content (Liu et al., 2018).

Studies have shown that PAL and cinnamic acid coenzyme A ligase (4CL) play a key role in lignin biosynthesis. PAL is the main rate-limiting enzyme that converts L-phenylalanine into trans-cinnamic acid by eliminating ammonia, with its isomers playing a unique but redundant role in plant growth and stress response (Bai et al., 2021). Moreover, 4CL acts as the main branch point enzyme controlling the biosynthesis of downstream molecules, particularly flavonoids and lignans. We identified that the PAL-encoding gene was upregulated under both HT35 and HT40 treatments, whereas those encoding 4CL enzymes were significantly more downregulated under HT40 than in the HT35 treatment. This hypothetically confirmed that higher HTS intensity better stimulated the phenylpropane pathway, allowing more lignin synthesis pathway-related genes. The lignin content in the Arabidopsis PAL gene quadruple mutant (*pal1*/*pal2*/*pal3*/*pal4*) was reduced by 20–25% compared to the wild type, with salicylic acid levels also being reduced (Huang et al., 2010). Cinnamoyl coenzyme A reductase (CCR) and cinnamyl alcohol dehydrogenase (CAD) are the last two enzymes that play a role in the mono-lignin synthesis pathway. Different isoforms of CCR have different functions, including defense, development, and stress, with their activity being positively correlated with lignin deposition in the xylem ducts (Li et al., 2013). Lowering CAD1 altered the lignin content and structure of poplar (Van Acker et al., 2017) while downregulating the CCR gene reduces the effectiveness of lignin C4 lactacystin in C4 forage (Giordano et al., 2014). In this study, the CCR and CAD genes were found to be upregulated under HT40 treatment, with significantly higher expression compared to HT35, consistent with the results of Gui et al. (2011). Therefore, studying the anatomy of raspberry leaves and stems under different HS treatments would be valuable to better highlight the importance of internal changes in the ductal tissue and tracheal structure for dispersing water transport in raspberries.

## 5 Conclusion

**Figure 12:**
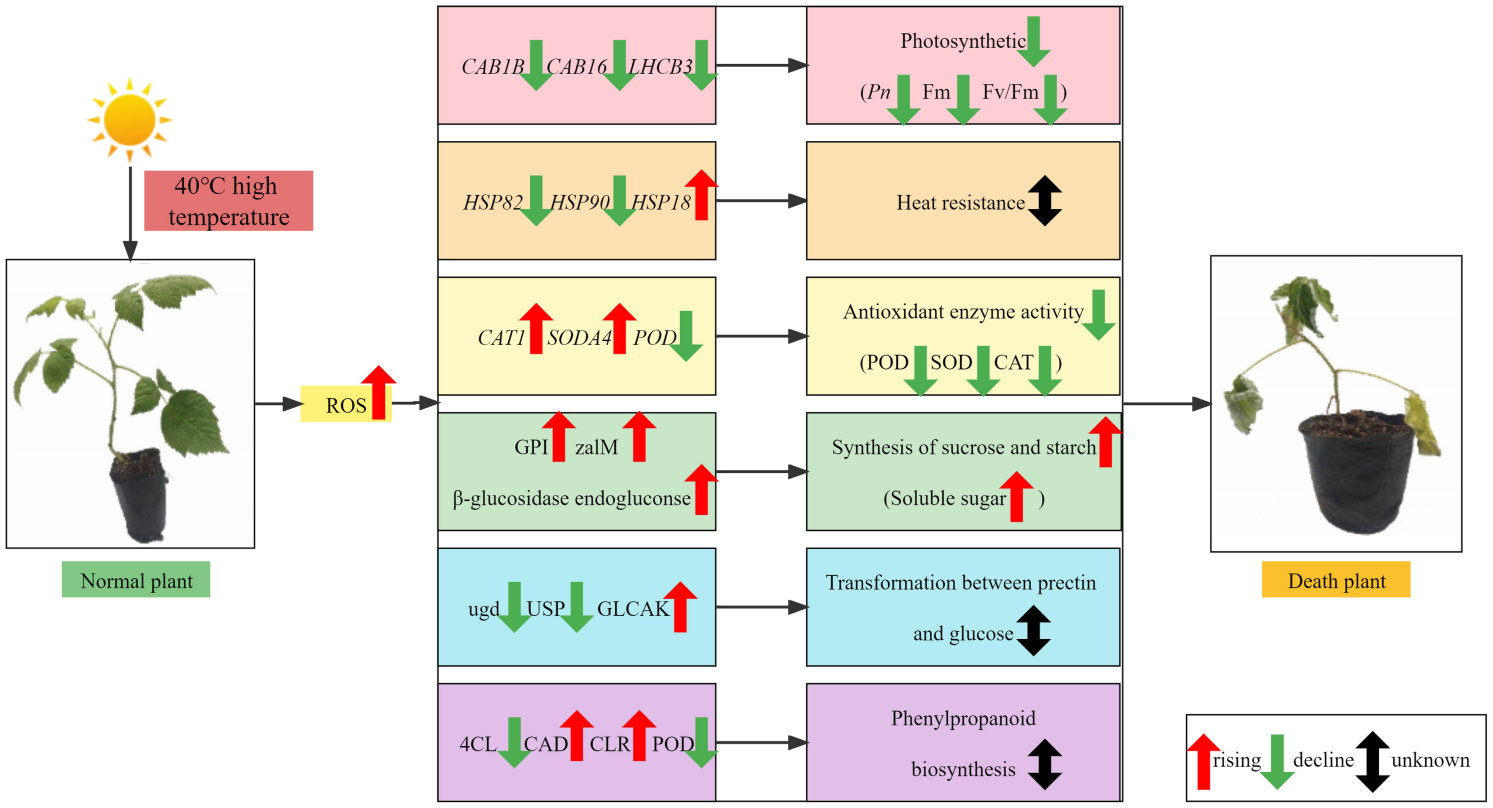
Regulatory model and key pathways of red raspberry seedling polka under HTS.

Therefore, a working model is proposed to explain the impact of HS on raspberry leaves (**Figure 12**). HTS induces excessive ROS accumulation in raspberry, causing oxidative damage to botanical cells and reducing photosynthesis. This, in turn, affects photosynthetic carbon fixation and starch and sucrose metabolism, which helps mitigate the HS-induced damage. Simultaneously, excessive ROS concentration promotes the activities of oxidant lyases (SOD and POD). With increasing stress duration, the chlorophyll content of red raspberry ‘Polka’ leaves initially decreased and then increased, except under severe stress, where it continued to decrease. The relative conductivity, MDA content, and soluble protein content of red raspberry leaves increased, while soluble sugar content initially decreased and then increased, CAT activity increased, SOD activity initially increased and then decreased, and POD activity gradually decreased with increasing HTS duration. Moreover, HT40 photosynthesis and fluorescence parameters showed lower values. Analysis of HTS identified 42 HSP family genes, two SOD-related DEGs, 25 POD-related DEGs, three CAT-related DEGs, 38 photosynthesis-related DEGs, etc. KEGG analysis showed that these DEGs were mainly enriched in pathways, including photosynthesis, photosynthesis-antennal protein, and phenylpropanoid biosynthesis. Although the expression of HTS-related plant genes was evaluated using physiological and transcriptomic analyses, regulating plant responses to HTS involves a complex regulatory network. The precise relationships between these genes and their distinct functions remain unclear and warrant deeper exploration.

## Data availability statement

The original contributions presented in the study are included in the article/**Supplementary Material**. Further inquiries can be directed to the corresponding author.

## Author contributions

JG and RY were responsible for writing the original manuscript. ZF conducted the research. JG and SC compiled the data. GQ, SG, PJ, and HL contributed to the resources. XZ and JG were responsible for funding acquisition and writing. XZ was responsible for funding acquisition and writing, reviewing, and editing. All authors contributed to the article and approved the submitted version.
