# Supplementary material for: Physiological and transcriptomic analysis reveal the crucial factors in heat stress response of red raspberry ‘Polka’ seedlings
Source: Front Plant Sci. 2023 Aug 9;14:1233448. doi: 10.3389/fpls.2023.1233448 (PMC10445156; doi:10.3389/fpls.2023.1233448)
Supplement: Supplementary file 1 [file DataSheet_1.zip › Date sheet_1/Supplementary_Material.docx]

Supplementary Material

# Supplementary Tables

**Supplementary Table 1.** Primers used in the study.

**Supplementary Table 2.** Filtered quality statistics

# Date sheet _1 and Date sheet _2: All raw data for the remaining figures.
